# Scavenger with Protonated Phosphite Ions for Incredible Nanoscale ZrO_2_-Abrasive Dispersant Stability Enhancement and Related Tungsten-Film Surface Chemical–Mechanical Planarization

**DOI:** 10.3390/nano11123296

**Published:** 2021-12-04

**Authors:** Seong-In Kim, Gi-Ppeum Jeong, Seung-Jae Lee, Jong-Chan Lee, Jun-Myeong Lee, Jin-Hyung Park, Jae-Young Bae, Jea-Gun Park

**Affiliations:** 1 Department of Nanoscale Semiconductor Engineering, Hanyang University, Seoul 04763, Korea; rlatjddls25805@naver.com (S.-I.K.); student.jongchan@gmail.com (J.-C.L.); kjunom200@naver.com (J.-M.L.); 2 Department of Electronic Engineering, Hanyang University, Seoul 04763, Korea; bono23231@naver.com (G.-P.J.); ltwobooml@naver.com (S.-J.L.); 3 UB Materials Inc., Yongin 17162, Korea; parkjinhyung@gmail.com; 4 Department of Energy Engineering, Hanyang University, Seoul 04763, Korea; jaeyoungb@daum.net

**Keywords:** tungsten, chemical-mechanical-planarization, scavenger, protonated phosphite-ion, Fenton reaction, corrosion

## Abstract

For scaling-down advanced nanoscale semiconductor devices, tungsten (W)-film surface chemical mechanical planarization (CMP) has rapidly evolved to increase the W-film surface polishing rate via Fenton-reaction acceleration and enhance nanoscale-abrasive (i.e., ZrO_2_) dispersant stability in the CMP slurry by adding a scavenger to suppress the Fenton reaction. To enhance the ZrO_2_ abrasive dispersant stability, a scavenger with protonate-phosphite ions was designed to suppress the time-dependent Fenton reaction. The ZrO_2_ abrasive dispersant stability (i.e., lower H_2_O_2_ decomposition rate and longer H_2_O_2_ pot lifetime) linearly and significantly increased with scavenger concentration. However, the corrosion magnitude on the W-film surface during CMP increased significantly with scavenger concentration. By adding a scavenger to the CMP slurry, the radical amount reduction via Fenton-reaction suppression in the CMP slurry and the corrosion enhancement on the W-film surface during CMP performed that the W-film surface polishing rate decreased linearly and notably with increasing scavenger concentration via a chemical-dominant CMP mechanism. Otherwise, the SiO_2_-film surface polishing rate peaked at a specific scavenger concentration via a chemical and mechanical-dominant CMP mechanism. The addition of a corrosion inhibitor with a protonate-amine functional group to the W-film surface CMP slurry completely suppressed the corrosion generation on the W-film surface during CMP without a decrease in the W- and SiO_2_-film surface polishing rate.

## 1. Introduction

Recently, nanoscale semiconductor devices have been rapidly scaling down to achieve faster switching, lower power consumption, and lower bit cost; that is, less than a 14 nm design rule for dynamic random-access memory (DRAM), higher than 128-floor memory-cells for three-dimensional (3D) NAND flash memory, and less than 5 nm design rule for application processors [1,2,3,4,5]. For fabricating advanced nanoscale semiconductor devices, tungsten (W)-film surface chemical mechanical planarization (CMP) utilized for W buried–gate, W wiring, and plugs has evolved toward a critical CMP performance, such as a higher W-film surface polishing rate, free of dishing, and erosion at a smaller W line width and a higher aspect ratio at the remaining W-film in plugs after CMP [6,7,8,9]. The W-film surface CMP is principally conducted by the Fenton reaction between the ferric–ionic catalyst and oxidant (i.e., hydrogen peroxide: H_2_O_2_) [10,11], which produces significantly dissolved O_2_ and radicals (i.e., OH^●^, HO_2_^●^ and O_2_^●−^) and chemically oxidizes the W film surface by forming a nanoscale thick oxide layer (i.e., WO_3_). Thus, the W-film surface CMP is conducted by mechanical rubbing between nanoscale abrasives (i.e., colloidal silica or ZrO_2_) and a nanoscale thick WO_3_ layer on the W-film surface. In particular, the produced radicals accelerate the Fenton reaction, which is called a cycling chemical reaction process [12,13]. Recently, several studies on W-film surface CMP have been reported, i.e., a design for a new ferric-ionic catalyst for enhancing the W-film surface polishing rate [11,14], a design for a selectivity agent for increasing the polishing rate selectivity between W- and SiO_2_-film surface [15], and a design for the scavenger for improving the abrasive stability in CMP slurry [10]. In particular, to enhance the W-film polishing rate during CMP, the Fenton reaction between a ferric–ionic catalyst and oxidant (i.e., H_2_O_2_) was essentially accelerated by designing ferric–ionic catalysts properly and increasing the oxidant concentration, which is a chemical-dominant CMP mechanism. However, the acceleration of the Fenton reaction in the W-film surface CMP slurry can induce a remarkable degradation of the nanoscale abrasive dispersant stability in the CMP slurry, resulting in fast sedimentation of the nanoscale abrasives in the CMP slurry during CMP [10].

Thus, to enhance the abrasive dispersant stability in the CMP slurry, a scavenger with carboxyl functional groups (i.e., trilithium citrate tetrahydrate: TCT–Li) for suppressing the Fenton reaction was proposed, which showed a H_2_O_2_ pot lifetime of less than 50 h. Note that the H_2_O_2_ pot lifetime is defined by the rapid increase in the secondary abrasive size after the oxidant (i.e., H_2_O_2_) was mixed with the W-film surface CMP slurry [10]. In addition, the achievement of a H_2_O_2_ pot lifetime longer than 7 days is vitally necessary for massive semiconductor fabrication. As a solution to further enhance the H_2_O_2_ pot lifetime, in this study, a scavenger with protonated phosphite or phosphate ions was designed for a W-film surface CMP slurry. First, the effect of the nanoscale ZrO_2_-abrasive dispersant stability on the scavenger type and concentration was examined, where two scavengers with protonated phosphite ions (i.e., etidronic acid: EA and phosphorous acid: PA) and two scavengers with protonated phosphate ions (i.e., monoammonium phosphate: MAP, monopotassium phosphate: MPP) were tested. Note that nanoscale ZrO_2_ abrasives were used for a W-film surface CMP slurry, since they could achieve a dishing-free W-film surface CMP performance [11]. The improvement in the magnitude of the ZrO_2_ abrasives in the slurry was estimated by the H_2_O_2_ decomposition rate and H_2_O_2_ pot lifetime. Thus, the dependence of the H_2_O_2_ decomposition rate on the scavenger type and concentration in the W-film surface CMP slurry was investigated. In particular, the Fenton reaction, depending on the scavenger type and concentration, was analyzed by the chemical reaction equations. In addition, the dependencies of the W- and SiO_2_-film surface polishing rate on the scavenger type and concentration were estimated. Moreover, the dependence of the W-film surface corrosion magnitude (i.e., static etch rate and corrosion current) on the scavenger concentration were observed to characterize the chemical-dominant CMP performance. Finally, the CMP mechanism (i.e., chemical-dominant CMP or mechanical-dominant CMP) of the W- and SiO_2_-film surface, depending on the scavenger concentration, was proved by chemical composition analysis of the W- and SiO_2_-film surfaces using X-ray photoelectron spectroscopy (XPS) and calculating the electrostatic force between the ZrO_2_ abrasive and the zeta potential of W- and SiO_2_-film surfaces.

## 2. Materials and Methods

### 2.1. Materials

A 300 nm-thick SiO_2_-film was deposited on a 12 inch-diameter Si wafer by chemical vapor deposition. The W-film surface CMP slurries were composed of colloidal monoclinic crystallized ZrO_2_ abrasives with a 40 nm primary abrasive size, a catalyst (i.e., Fe(NO_3_)_3_), a phosphite-based scavenger (i.e., etidronic acid: EA or phosphorous acid: PA) or a phosphate-based scavenger (i.e., monopotassium phosphate: MPP or monoammonium phosphate: MAP), a corrosion inhibitor (i.e., asparagine), a pH titrant (i.e., HNO_3_), an oxidant (i.e., H_2_O_2_), and deionized water (DIW). The ZrO_2_ abrasives (1 wt%) were dispersed using a polycarboxylic acid-type dispersant through a ball-mill process in DIW. Phosphite- or phosphate-based scavengers (0–0.25 wt%) were added to the W-film surface CMP slurries. Ferric nitrate (i.e., Fe(NO_3_)_3_) of 0.15 wt% was used as a Fenton reaction catalyst to increase the W-film surface polishing rate. Afterward, the slurries were titrated to pH 2 using a pH titrant (i.e., HNO_3_). The slurry and DIW ratios were diluted to 1:1, and then 1.5 wt% of H_2_O_2_ was added to the W-film surface CMP slurries.

### 2.2. CMP Conditions

To estimate the CMP performance of the slurries, a 12 inch diameter wafer with a vertical structure of Si substrate, 100 nm thick SiO_2_ film, 100 nm thick TiN film and 250 nm thick W-film, and a 12 inch diameter wafer with a vertical structure of Si substrate, a 450 nm thick SiO_2_ film were used. The wafers were polished using a polishing machine (AP-300, CTS Co., Inc., Cheongju, Korea) and a CMP pad (IC 1000/Suba IV, Dupont Co., Inc., Wilmington, DE, USA). The W-film surface slurries were diluted with DIW to a ratio of 1:1. After that, H_2_O_2_ was added to the CMP slurry, and the mixed slurry was stirred at 300 rpm for 1 h in the CMP supply tank. The mixed slurries with a slurry flow rate of 200 mL/min were dropped from the supply tank over the CMP pad during CMP. The polishing table rotation speed and wafer carrier rotation speed were 87 and 93 rpm, respectively, the polishing head pressure was 3 psi, and the polishing time was 1 min.

### 2.3. Measurement Equipment

The polishing rate of the SiO_2_-film surface was calculated by measuring the SiO_2_-film thickness before and after CMP using an ellipsometer (V-VASE, J.A. Woollam Co., Inc., Lincoln, NE, USA). In addition, the W-film surface polishing rate was estimated by measuring the difference in sheet resistance before and after CMP using a four-point probe (CMT-SR5000, AIT Co., Inc., Suwon, Korea). After dipping the W film into W-film CMP slurries at 65 °C for 3 min, the surface morphology of the W-film surfaces was observed using a scanning electron microscope (SEM, S-4800, Hitachi Co., Inc., Tokyo, Japan) at an accelerating voltage of 5 kV. A potentiostat (CHI750, CH Instruments Co., Inc., Austin, TX, USA) was used to measure the corrosion potential and current of the W-film surface after dipping the W film into W-film CMP slurries at 65 °C for 5 min. The chemical composition of the W-film surface after CMP was analyzed by X-ray photoelectron spectroscopy (XPS, K-Alpha+, Thermo Fisher Scientific Co., Inc., Waltham, MA, USA) using an Al Kα source (1486.6 eV) at 12 KeV and 6 mA. The secondary abrasive size was measured using a laser-scattering particle size distribution analyzer (LA-960S, HORIBA Scientific Co., Inc., Kyoto, Japan) to estimate the ZrO_2_ abrasive dispersant stability in the W-film CMP slurry. The zeta potentials of the ZrO_2_ abrasives, WO_3_ particles, and SiO_2_ particles were measured with a particle analyzer using the electrophoretic light-scattering method (ELSZ2+, Otsuka Electronics Co., Inc., Osaka, Japan).

## 3. Results and Discussion

### 3.1. Abrasive Dispersant Stability of W-Film Surface CMP Slurry Depending on Phosphite or Phosphate-Based Scavenger Type and Concentration

The Fenton reaction between ferric–ionic catalyst (i.e., Fe(NO_3_)) and oxidant (i.e., H_2_O_2_) including a phosphite-based scavenger (i.e., C_2_H_8_O_7_P_2_: EA) can be described by Equations (1)–(11).

Fe(NO_3_)_3_ → Fe^3+^ + 3(NO_3_)^−^ (1)

Fe^3+^ + H_2_O_2_ → FeOOH^2+^ + H^+^ (2)

FeOOH^2+^ → Fe^2+^ + HO_2_^●^ (3)

Fe^2+^ + H_2_O_2_ → Fe^3+^ + OH^−^ + OH^●^ (4)

OH^●^ + H_2_O_2_ → H_2_O + HO_2_^●^ (5)

HO_2_^●^ → H^+^ + O_2_^●−^ (6)

O_2_^●−^ + Fe^3+^ → O_2_ + Fe^2+^ (7)

C_2_H_8_O_7_P_2_ → 2H^+^ + (C_2_H_6_O_7_P_2_)^2−^ (8)

4OH^●^ + 2(C_2_H_6_O_7_P_2_)^2−^ → 4H_2_O + (C_2_H_7_O_7_P_2_)-(C_2_H_7_O_7_P_2_) (9)

4HO_2_^●^ + 2(C_2_H_6_O_7_P_2_)^2−^ → 2O_2_ + 4H_2_O + (C_2_H_7_O_7_P_2_)-(C_2_H_7_O_7_P_2_) (10)

2O_2_^●−^ + 4(C_2_H_6_O_7_P_2_)^2−^ → 4H_2_O + 2[(C_2_H_7_O_7_P_2_)-(C_2_H_7_O_7_P_2_)] (11)

6OH^●^ + W → WO_3_ + 3H_2_O (12)

W + O_2_ → WO_2_ (13)

2WO_2_ + O_2_ → 2WO_3_ (14)

2WO_3_ + 2(C_2_H_6_O_7_P_2_)^2−^ + 3O_2_ → 2(WO_4_^2−^) + 4CO_2_ + 4H_2_O + 2H_2_PO_3_^−^ (15)

**Figure 1:**
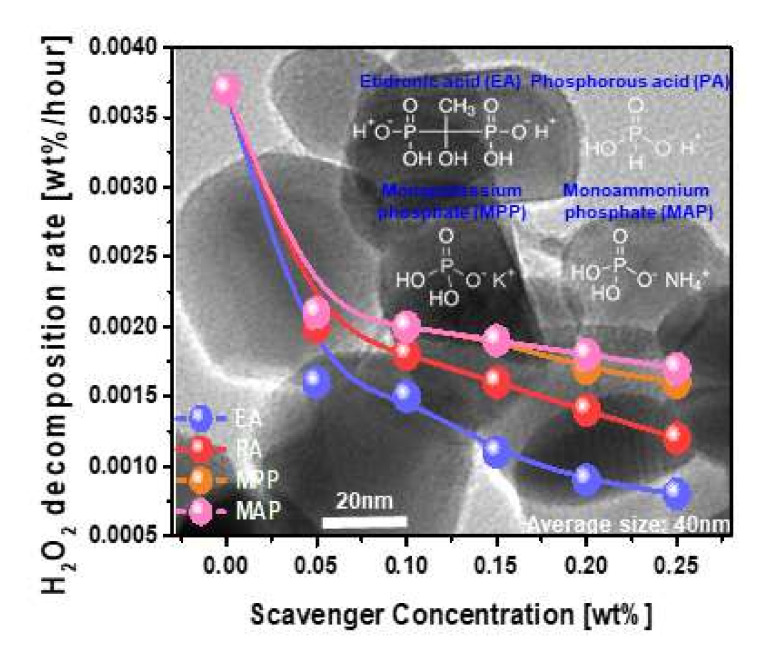
Dependency of the H_2_O_2_ decomposition rate on the scavenger type and concentration.

The Fenton reaction produces reactive radicals such as OH^●^, HO_2_^●^, and O_2_^●−^ in the W-film surface CMP slurry, and the produced OH^●^, HO_2_^●^, and O_2_^●−^ significantly accelerated the decomposition of H_2_O_2_ into the dissolved O_2_ in the slurry; this process is called a cycling chemical reaction process, as shown in Equations (1)–(7). As a result, the W-film surface was chemically oxidized; that is, the formation of WO_3_ on the W-film surface, as shown in Equations (12)–(14). The W-film surface CMP is principally conducted by the formation of WO_3_ on the W-film surface, followed by mechanical rubbing between the ZrO_2_ abrasive and the nanoscale thick WO_3_ layer. However, the produced OH^●^, HO_2_^●^, and O_2_^●−^ could chemically react with the dispersant polymer (i.e., polycarboxylic acid-type polymer) coated on ZrO_2_ abrasives, which significantly degrades the ZrO_2_ abrasive dispersant stability in the slurry [16,17]. The addition of a phosphite-based scavenger (i.e., etidronic acid (C_2_H_8_O_7_P_2_): EA) to the W-film surface CMP slurry significantly suppressed the Fenton reaction via a chemical reaction between the negatively charged C_2_H_6_O_7_P_2_^2−^ and radicals (i.e., OH^●^, HO_2_^●^ and O_2_^●−^), producing H_2_O_2_ and neutral C_2_H_8_O_7_P_2,_ as shown in Equations (8)–(11). As a result, the amount of OH^●^, HO_2_^●^ and O_2_^●−^ in the slurry could be reduced significantly, improving the stability of the ZrO_2_ abrasive dispersant in the slurry. Thus, in order to estimate the magnitude of improvement of the ZrO_2_ abrasive dispersant stability in the W-film surface CMP slurry including a scavenger, the dependence of the H_2_O_2_ decomposition rate on a phosphite (i.e., EA and PA) or a phosphate (i.e., MPP and MAP)-based-scavenger type and concentration were investigated since the H_2_O_2_ decomposition rate could represent the magnitude of the ZrO_2_ abrasive dispersant stability in the slurry, as shown in Figure 1. EA, PA, MPP, and MAP correspond to etidronic acid with double-negatively charged phosphite ions, phosphorous acid with a single negatively charged phosphite ion, monopotassium phosphate with a single negatively charged phosphate ion, and monoammonium phosphate with a single negatively charged phosphate ion, respectively, as shown by the chemical symbols in Figure 1. The H_2_O_2_ decomposition rate (i.e., H_2_O_2_ concentration/h) was estimated by measuring the slope of the remaining H_2_O_2_ concentration to the progress time after H_2_O_2_ was added to the slurry (i.e., the progress time), as shown in Figure S1. Note that the H_2_O_2_ concentration for all CMP slurries, including a scavenger, decreased when the progress time increased, and the slope of the remaining H_2_O_2_ concentration to the progress time indicates the H_2_O_2_ decomposition rate. In addition, a lower H_2_O_2_ decomposition rate implies a higher improvement in the stability of the ZrO_2_ abrasive dispersant in the W-film surface CMP slurry. For all four different phosphite or phosphate-based-scavengers, the H_2_O_2_ decomposition rate decreased rapidly from 0.0037 wt%/h to less than 0.0020 wt%/h as soon as a scavenger of 0.05 wt% was added to the W-film surface CMP slurry, and the H_2_O_2_ decomposition rate decreased linearly with increasing scavenger concentration, as shown in Figure 1. In addition, a lower sequence of the H_2_O_2_ decomposition rate at 0.25 wt% scavenger concentration was presented by EA (i.e., 0.0008 wt%/h), PA (i.e., 0.0012 wt%/h), MPP (i.e., 0.0016 wt%/h), and MAP (i.e., 0.0017 wt%/h), indicating that the higher sequence of the improvement effect of the ZrO_2_ abrasive dispersant stability in the slurry was followed by EA, PA, MPP, and MAP. In particular, the higher sequence of the decrease in the slope of the H_2_O_2_ decomposition rate to the scavenger concentration (i.e., Fenton-reaction ability) was followed by EA (i.e., 0.0097 H_2_O_2_ wt%/h/EA wt%), PA (i.e., 0.0083 H_2_O_2_ wt%/h/EA wt%), MPP (i.e., 0.0067 H_2_O_2_ wt%/h/EA wt%), and MAP (i.e., 0.0063 H_2_O_2_ wt%/h/EA wt%), as shown in Figure 1. The dependency of the H_2_O_2_ decomposition rate on the molecular structure of the scavengers was related to the mole concentration and the dissociation constant of protonated phosphite or phosphate ions, as shown in Figure S2. There were two groups of scavengers, i.e., those with protonated phosphite ions (i.e., EA and PA) and those with phosphate ions (i.e., MPP and MAP). At the same scavenger mole concentration, the effect of the scavengers with protonated phosphite ions (i.e., H_2_O_2_ decomposition rate) was better than that of those with protonated phosphate ions. Among scavengers with protonated phosphite ions, at the same scavenger mole concentration, EA showed a higher scavenger effect than PA, since EA contained two protonated phosphite ions while PA had one protonated phosphite ion. Otherwise, among the scavengers with protonated phosphate ions, MPP showed a slightly higher scavenger effect than MAP, since the dissociation of the protonated phosphate ion in MAP (i.e., NH_4_^+^) was much more difficult than that in MPP (i.e., K^+^). Note that the dissociation constant of NH_4_^+^ in MAP was K_a_ = 1.8×10^−5^. This result also indicates that a higher sequence of the ZrO_2_ abrasive dispersant stability improvement effect was achieved by EA, PA, MPP, and MAP. In summary, the addition of a phosphite or a phosphate-based scavenger to the W-film surface CMP slurry significantly reduced the H_2_O_2_ decomposition rate, which clearly improved the ZrO_2_ abrasive dispersant stability in the slurry. In addition, the H_2_O_2_ decomposition ratio decreased linearly with the scavenger concentration, so that the improvement in the magnitude of the ZrO_2_ abrasive dispersant stability in the slurry would be enhanced by increasing the scavenger concentration. Moreover, the magnitude of improvement of the ZrO_2_ abrasive dispersant stability in the slurry strongly depended on the scavenger type; that is, a higher sequence of the improvement magnitude of the ZrO_2_ abrasive dispersant stability in the slurry was followed by EA, PA, MPP, and MAP.

### 3.2. Dependency of W- and SiO_2_-Film Polishing Rate on Scavenger Type and Concentration

**Figure 2:**
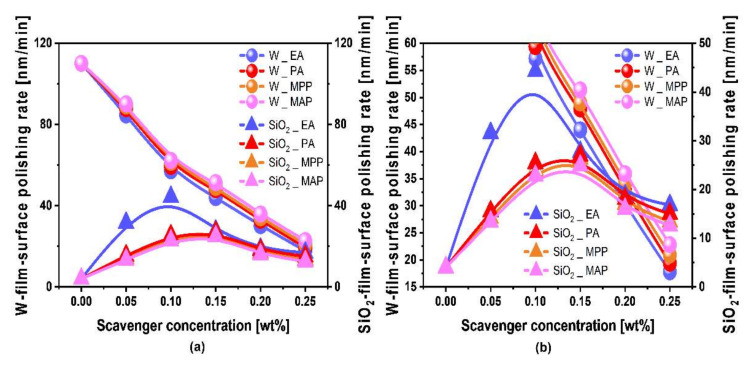
CMP performance of W-film surface slurries. (**a**) W- and SiO_2_-film surface polishing rates depending on the scavenger type and concentration; (**b**) magnified W- and SiO_2_-film surface polishing rates from (**a**).

To estimate the influence of the chemical properties of the scavenger on the chemical–mechanical-polishing performance of W- and SiO_2_-film surfaces, the W- and SiO_2_-film surface polishing rates were investigated as a function of the scavenger type (i.e., EA, PA, MPP, and MAP) and concentration in the CMP slurries, as shown Figure 2. For all W-film surface CMP slurries including a scavenger, the W-film surface polishing rate decreased linearly and rapidly from ~110.1 to ~17.8 nm/min when the scavenger concentration increased from 0 to 0.25 wt%. To distinguish the detailed W-film surface polishing rate among scavengers, the magnified W-film surface polishing rate vs. scavenger concentration in Figure 2a was magnified, as shown in Figure 2b. The lower sequence of the W-film surface polishing rate at the same scavenger concentration was clearly presented by EA, PA, MPP, and MAP, although their difference was less. The dependency of the H_2_O_2_ decomposition rate on the scavenger type in Figure 1 was well correlated with that of the W-film surface polishing rate (Figure 2b); that is, a lower H_2_O_2_ decomposition rate led to a lower W-film surface polishing rate. A lower H_2_O_2_ decomposition rate produced a lower magnitude of chemical oxidation (i.e., WO_3_) on the W film surface via the Fenton reaction during CMP, as shown in Equations (1)–(12). Thus, since the lower sequence of the H_2_O_2_ decomposition rate was followed by EA, PA, MPP, and MAP, the lower sequence of the W-film surface polishing rate was presented by EA, PA, MPP, and MAP, as shown in Figure 2b.

Otherwise, for all W-film surface CMP slurries including a scavenger, the SiO_2_-film surface polishing rate increased significantly with the scavenger concentration up to a specific scavenger concentration and then decreased remarkably with increasing scavenger concentration. This result indicates that the SiO_2_-film polishing rate peaks at a specific scavenger concentration. The mechanism by which the SiO_2_-film polishing rate peaks at a specific scavenger concentration will be discussed later. The scavenger concentrations showing a peaked SiO_2_-film surface polishing rate for the CMP slurries including EA, PA, MPP, and MAP were 0.10, 0.15, 0.15, and 0.15 wt%, respectively. The higher sequence of the peaked SiO_2_-film surface polishing rate for the CMP slurries including a scavenger was presented by EA (i.e., 44.3 nm/min), PA (i.e., 27.0 nm/min), MPP (i.e., 26.3 nm/min), and MAP (i.e., 24.9 nm/min), as shown in Figure 2b. The dependency of the peaked SiO_2_-film surface polishing rate on the scavenger type as shown in Figure 1 is also well correlated with that of the H_2_O_2_ decomposition rate as shown in Figure 2b; that is, a lower H_2_O_2_ decomposition rate led to a higher SiO_2_-film surface polishing rate. Since a lower H_2_O_2_ decomposition rate generates lower dissolved O_2_ and radicals (i.e., OH^●^, HO_2_^●^, and O_2_^●−^), it would perform as a higher SiO_2_-film surface polishing rate because of chemical oxidation between the Si(OH)_4_-film surface and radicals (i.e., OH^●^, HO_2_^●^, and O_2_^●−^). Note that the SiO_2_-film surface would be transformed from SiO_2_ to Si(OH)_4_ via a hydrolysis reaction in the CMP slurry. Thus, since a lower sequence of the H_2_O_2_ decomposition rate in the slurry was presented by EA, PA, MPP, and MAP, a higher sequence of SiO_2_-film surface polishing rate was shown by EA, PA, MPP, and MAP.

### 3.3. Dependencies of Chemical Properties (i.e., Corrosion, Potentiodynamic Polarization, and Chemical Composition) on Scavenger (i.e., EA) Concentration

**Figure 3:**
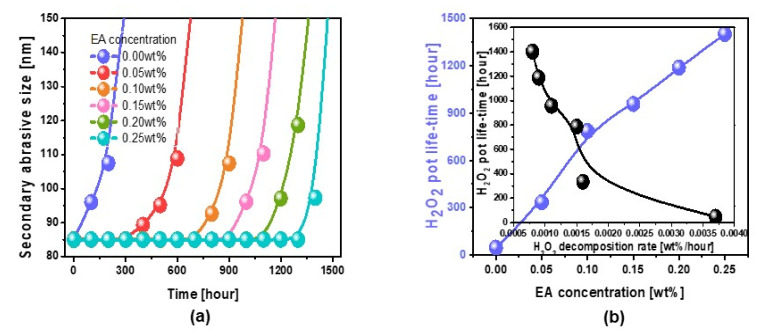
Effect of scavenger (i.e., EA) on the ZrO_2_ abrasive stability in W-film surface slurries mixed with 1.5 wt% H_2_O_2_. (**a**) Secondary abrasive size vs. the progress time after mixing H_2_O_2_ into the W-film surface slurries, depending on the scavenger (i.e., EA) concentration. (**b**) H_2_O_2_ pot lifetime depending on the scavenger concentration. The inset represents the correlation between H_2_O_2_ pot lifetime and H_2_O_2_ decomposition rate.

To understand the mechanism of the dependencies of the W- and SiO_2_-film surface polishing rate on the scavenger concentration, etidronic acid with double-negatively charged phosphite ions (i.e., EA) was selected. Again, the dependence of the abrasive dispersant stability on the scavenger concentration (i.e., EA) was investigated by measuring the time-dependent secondary abrasive size in the W-film surface CMP slurries after mixing H_2_O_2_ with the CMP slurry, as shown in Figure 3a. For all CMP slurries with different scavenger concentrations, the secondary abrasive size rapidly increased with the progress time after mixing H_2_O_2_ with the CMP slurry at a specific time (called the H_2_O_2_ pot lifetime). However, the pot lifetime increased linearly from 47 to 1401 h when the scavenger concentration increased from 0 to 0.25 wt%, as shown in Figure 3b. This result was well correlated with the H_2_O_2_ decomposition rate (Figure 1); that is, the H_2_O_2_ pot lifetime increased exponentially when the H_2_O_2_ decomposition rate decreased. Surprisingly, the H_2_O_2_ pot lifetime enhanced from 47 to 335 h, although the EA of 0.05 wt% was added to the W-film surface CMP slurry, indicating that the addition of the scavenger (i.e., EA) is very effective for achieving a better ZrO_2_ abrasive dispersant stability in the slurry.

**Figure 4:**
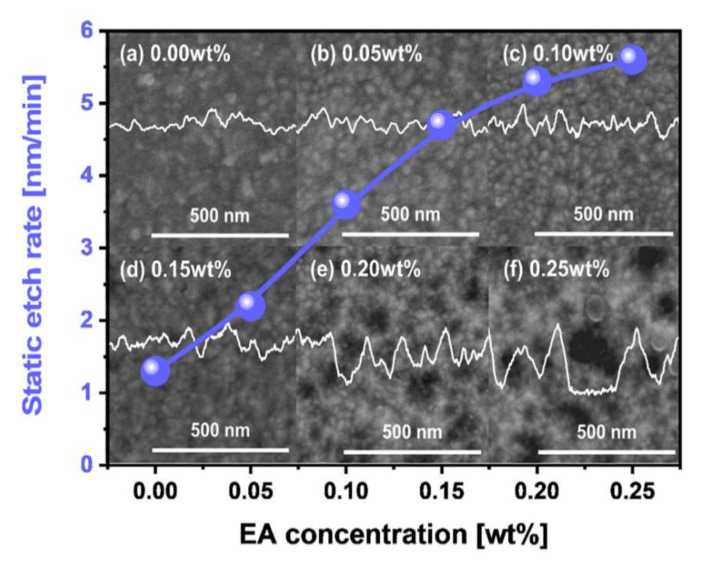
Corrosion magnitude (i.e., static etch rate) and corrosion-induced surface morphology depending on the scavenger (i.e., EA) concentration. (**a**) EA of 0.00 wt%; (**b**) EA of 0.05 wt%; (**c**) EA of 0.10 wt%; (**d**) EA of 0.15 wt%; (**e**) EA of 0.20 wt%; (**f**) EA of 0.25 wt%. Background SEM images show the presence and magnitude of corrosion on the W-film surface after dipping the W-film into the CMP slurry with a scavenger (i.e., EA). The lateral profiles of image contrast on the SEM images correspond to the magnitude of corrosion.

To understand how the addition of a scavenger to the W-film surface CMP slurry affects the morphology of the W-film surface, the dependencies of the magnitude of corrosion and surface morphology on the scavenger concentration were estimated by measuring the static etch rate at 65 °C for 3 min and by observing the SEM images after etching, as shown in Figure 4. The static etch rate (i.e., magnitude of corrosion) increased significantly from 1.3 to 5.6 nm/min when the scavenger (i.e., EA) concentration increased from 0 to 0.25 wt%. Note that the reason for the corrosion (i.e., etching) on the W-film surface via the addition of the scavenger (i.e., EA) could be understood by considering the chemical reaction between WO_3_, (C_2_H_6_O_7_P_2_)^2−^, and O_2_, producing WO_4_^2−^, CO_2_, H_2_O, and H_2_PO_3_^−^ as shown in Equation (15). In addition, the surface morphology induced by corrosion increased significantly with the scavenger concentration, as shown in the background SEM images in Figure 4. This result indicates that the addition of a scavenger (i.e., EA) to the W-film surface CMP slurry could etch the W-film surface so that the W-film surface roughness was remarkably enhanced by etching at the grain boundaries of the poly-W-film surface. Thus, the size of the corrosion-induced pits increased notably with the scavenger concentration, as shown in the lateral line profiles of the surface roughness in Figure 4.

**Figure 5:**
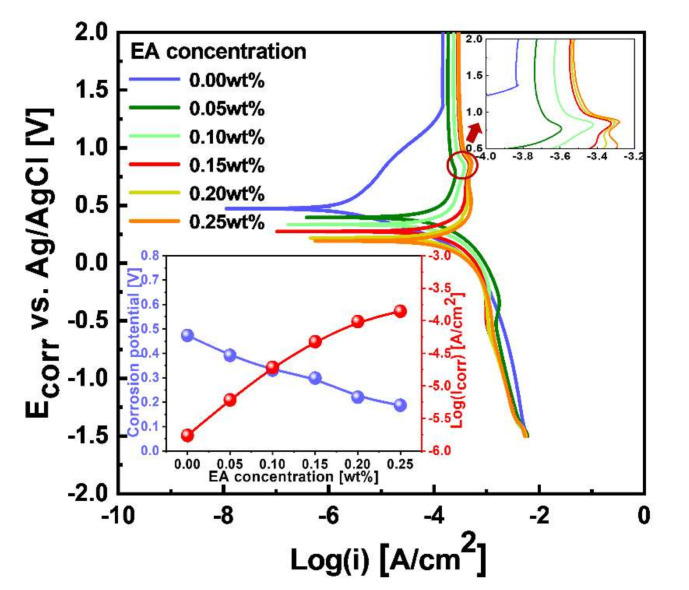
Potentiodynamic polarization curves of the W-film surface depending on the scavenger (i.e., EA) concentration after dipping the W-film surface into the W-film surface slurries. The upper inset represents the chemical oxidation magnitude on the W-film surface. The lower inset indicates dependencies of corrosion potential (i.e., E_corr_) and corrosion current (i.e., I_corr_) depending on the scavenger (i.e., EA) concentration.

To characterize how the addition of the scavenger (i.e., EA) to the W-film surface CMP slurry influences the magnitude of corrosion, the potentiodynamic polarizations of the W-film surfaces were observed as a function of the scavenger concentration, as shown in Figure 5. The presence of a porous chemical oxide layer on the W-film surface was found at the anode curve of the potentiodynamic polarization curve, as shown in the circle of Figure 5. The formation magnitude of the porous chemical oxide layer increased with the scavenger concentration, as shown in the magnified figure of the anode curve (i.e., the upper inset of Figure 5). From the potentiodynamic polarization curve, the dependencies of the corrosion potential (E_corr_) and corrosion current (I_corr_) were calculated using the Tafel method, as shown in the inset of Figure 5 [18]. E_corr_ decreased linearly from 0.47 to 0.19 V while I_corr_ increased significantly and linearly from 10^−5.8^ to 10^−3.9^ A/cm^2^, when the scavenger concentration increased from 0 to 0.25 wt%, meaning that the formation magnitude of porous WO_3_ layer on the W-film surface was noticeably enhanced with the scavenger concentration. These results imply that the addition of a scavenger to the W-film surface CMP slurry could enhance the magnitude of corrosion (i.e., formation magnitude of the porous chemical oxide layer). In addition, the dependence of the static etch rate on the scavenger concentration was well correlated with that of the I_corr_ on the scavenger concentration; that is, both I_corr_ and the static etch rate increased linearly and significantly with the scavenger concentration. Thus, the W-film surface polishing rate decreased linearly and significantly with the scavenger concentration, since the W-film surface polishing rate generally decreased with both the static etch rate and the formation magnitude of the porous oxide layer on the W-film surface [10].

**Figure 6:**
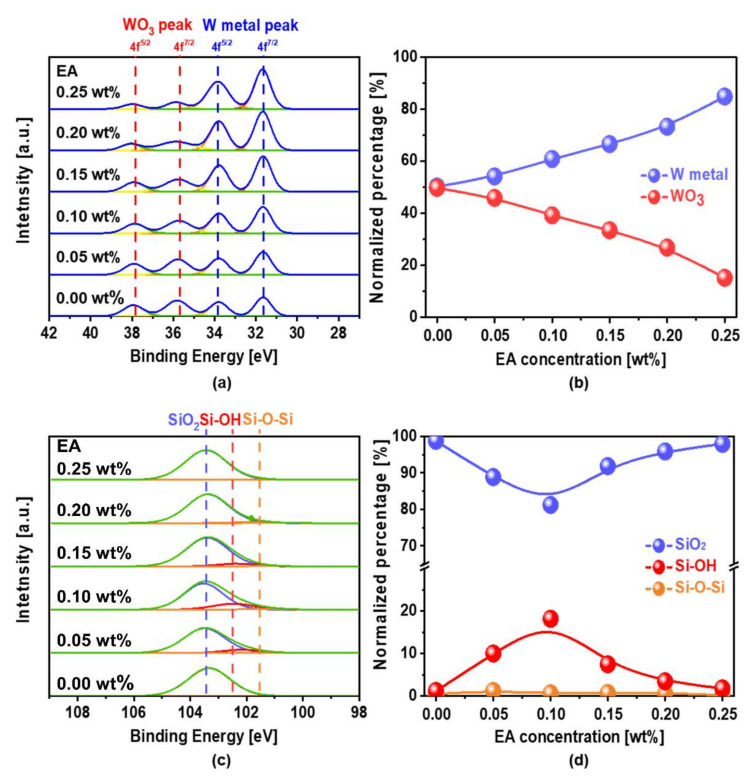
Chemical composition of W- and SiO_2_-film surfaces depending on the scavenger (i.e., EA) concentration and analyzed by XPS after the W-film surface CMP, depending on the scavenger (i.e., EA) concentration, (**a**) Spectra XPS intensity vs. binding energy of WO_3_ and W-metal on the W-film surface; (**b**) normalized XPS peak percentage of WO_3_ and W-metal on the W-film surface; (**c**) spectra intensity vs. binding energy of Si–O–Si, SiO_2_, and Si–OH on the SiO_2_-film surface, and (**d**) normalized XPS peak percentage of Si–O–Si, SiO_2_, and Si–OH on the SiO_2_-film surface after CMP.

To confirm the dependence of the chemical oxidation magnitude of the W-film surface on the scavenger concentration, the chemical composition (i.e., relative intensity of W-metal and WO_3_) of the W-film surface was analyzed as a function of the scavenger concentration using XPS. Reminders that the W-film surface could be chemically oxidized, as shown in Equations (13)–(14). In XPS, W metal 4f 7/2 and 4f 5/2 peaks were found at 31.6 and 33.8 eV, and those of WO_3_ peaks are located at 35.6 and 37.8 eV [19,20], respectively, as shown in Figure 6a. The normalized XPS-peak intensity at WO_3_ decreased almost linearly with increasing scavenger concentration, as shown in Figure 6b. Otherwise, the normalized XPS-peak intensity at the W-metal increased almost linearly with the scavenger concentration. These results indicate that the addition of a scavenger to the W-film surface CMP slurry evidently reduced the chemical oxidation magnitude (i.e., WO_3_) on the W-film surface; that is, the chemical oxidation magnitude on the W-film surface decreased clearly with increasing scavenger concentration. The dependence of the normalized XPS-peak intensity of WO_3_ on the W-film surface on the scavenger concentration was well calibrated with those of the static etch rate (i.e., corrosion) and corrosion current (i.e., I_corr_) on the scavenger concentration; that is, a lower normalized XPS-peak intensity of WO_3_ corresponded to a higher static etch rate and I_corr_. Moreover, the dependence of the hydrolysis magnitude of the SiO_2_-film surface on the scavenger concentration was observed as a function of the scavenger concentration using XPS, as shown in Figure 6c. The XPS-peak intensities of Si–O–Si, Si–OH, and SiO_2_ on the SiO_2_-film surface were presented at 101.5, 102.3, and 103.4 eV, respectively [21,22]. Note that the normalized intensity of Si–OH (i.e., hydrolysis magnitude) determines the SiO_2_-film polishing rate [10]; that is, a higher normalized intensity of Si–OH leads to a higher SiO_2_-film polishing rate, since chemical-dominant CMP was conducted by rubbing ZrO_2_ abrasives and a nanoscale thick Si–OH layer on the SiO_2_-film surface. After CMP, the normalized XPS-peak intensity of Si–OH on the SiO_2_-film surface peaked at a scavenger concentration of 0.10 wt%, while that of SiO_2_ on the SiO_2_-film surface was minimized at the same scavenger concentration. These results indicate that the normalized XPS-peak intensity of Si–OH on the SiO_2_-film surface depending on the scavenger concentration was well correlated with the SiO_2_-film polishing rate depending on the scavenger concentration. A higher normalized XPS-peak intensity of Si–OH on the SiO_2_-film surface led to a higher SiO_2_-film polishing rate. However, since the chemical properties (i.e., the amount of dissolved O_2_ and radicals) decreased linearly with increasing scavenger concentration, the normalized XPS-peak intensity of Si–OH on the SiO_2_-film surface depending on the scavenger concentration could not be directly understood by considering only a chemical-dominant CMP mechanism.

### 3.4. Dependency Mechanism of W- and SiO_2_-Film Surface Polishing Rates on Scavenger Concentration

**Figure 7:**
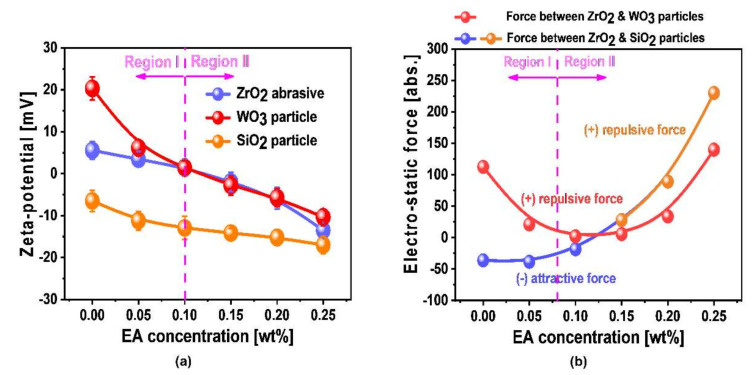
Dependencies of mechanical-dominant CMP properties on the scavenger concentration (i.e., EA). (**a**) Zeta potentials of ZrO_2_ abrasives, WO_3_ particles, and SiO_2_ particles depending on the scavenger (i.e., EA) concentration, and (**b**) electrostatic forces between ZrO_2_ abrasives and WO_3_ particles as well as between ZrO_2_ abrasives and SiO_2_ particles.

In general, both W- and SiO_2_-film surface polishing rates are principally determined by both chemical- and mechanical-dominant CMP. The hardness of the W-film surface (i.e., 6.64 GPa) is significantly lower than the SiO_2_-film surface (i.e., 11.62 GPa), as shown in Figure S3, revealing that the W-film surface polishing rate is determined by chemical-dominant CMP (i.e., corrosion and magnitude of chemical oxidation) rather than a mechanical-dominant CMP (i.e., electrostatic force between the abrasives and the W-film surface). Since the normalized XPS-peak intensity of the chemical oxidation magnitude (i.e., WO_3_) decreased significantly with increasing scavenger (i.e., EA) concentration and both the static etch rate (i.e., magnitude of corrosion) and corrosion current increased significantly with the scavenger concentration, the W-film surface polishing rate decreased remarkably with increasing scavenger concentration. To confirm that the W-film surface polishing rate is determined by a chemical-dominant CMP rather than a mechanical-dominant CMP, the dependence of the electrostatic force between the ZrO_2_ abrasive and WO_3_ layer on the W-film surface was estimated as a function of the scavenger (i.e., EA) concentration, as shown in Figure 7a. Note that the zeta potential of the WO_3_ layer and W-film surface was measured by the zeta potential of WO_3_ particles in the W-film surface CMP slurry, since the zeta potential of the W-film surface could not be directly measured by a particle analyzer because of the metallic characteristic of the W-film surface. Thus, the zeta potential of the WO_3_ particles responds to that of the WO_3_ layer on the W-film surface. The zeta potential of ZrO_2_ abrasives decreased from +5.5 to +1.4 mV when the scavenger concentration increased from 0 to 0.10 wt% and then it changed from a positive to a negative zeta potential and negatively increased significantly from +1.4 to −13.5 mV for further increase in scavenger-concentration. The zeta potential of the W-film surface decreased from +20.3 to +1.4 mV when the scavenger concentration increased from 0 to 0.10 wt%. Then, it transited from a positive to a negative zeta potential and negatively increased considerably from +1.4 to −10.4 mV for further increase in scavenger-concentration. As a result, the repulsive electrostatic force between the ZrO_2_ abrasive and the W-film surface decreased significantly from 112.5 to 2.0 abs. up to the EA of 0.10 wt% and then it increased remarkably for further increase in scavenger concentration, as shown in Figure 7b. Thus, if the W-film surface polishing rate is principally determined by a mechanical-dominant CMP, it would increase with the scavenger concentration increasing up to the EA of 0.10 wt% and then decreased with the scavenger concentration for further increase in EA-concentration; that is, the W-film polishing rate should be peaked at the EA of 0.10 wt%. Note that a higher repulsive electrostatic force results in a lower mechanical polishing rate. Therefore, since the W-film surface polishing rate decreased significantly with increasing scavenger (i.e., EA) concentration, the W-film surface CMP mechanism would be principally performed by chemical-dominant CMP rather than mechanical-dominant CMP.

In order to test whether the mechanism of the SiO_2_-film surface CMP is a chemical-dominant CMP or a mechanical-dominant CMP, the influence of the Fenton reaction in the CMP slurry including a scavenger was reviewed by considering Equations (16)–(19).

SiO_2_ + 2H_2_O → Si(OH)_4_ (16)

Si(OH)_4_ + 4OH^●^ → SiO_2_ + H_2_O + O_2_ (17)

2Si(OH)_4_ + 2O_2_^●−^ → 2SiO_2_ + 4H_2_O + 2O_2_ (18)

3Si(OH)_4_ + 4HO_2_^●^ → 3SiO_2_ + 8H_2_O + 3O_2_ (19)

Since a lower H_2_O_2_ decomposition rate produced a lower amount of dissolved O_2_ and radicals (i.e., OH^●^, HO_2_^●^ and O_2_^●−^) via the Fenton reaction, the SiO_2_-film surface would be chemically oxidized rather than chemically etched, as shown in Equations (16)–(19). Note that the SiO_2_-film surface transformed the Si(OH)_4_-film surface via a hydrolysis reaction, as shown in Equation (16), and then the Si(OH)_4_-film surface is oxidized via a chemical reaction with radicals (i.e., OH^●^, HO_2_^●^ and O_2_^●−^) by producing H_2_O_2_ and O_2_, as shown in Equations (17)–(19). Note that a higher chemical oxidation magnitude leads to a less SiO_2_-film surface polishing rate. Thus, a lower H_2_O_2_ decomposition rate corresponding to a higher scavenger (i.e., EA) concentration leads to a higher SiO_2_-film surface polishing rate. However, the SiO_2_-film surface polishing rate peaked at a specific scavenger concentration (i.e., EA of 0.10 wt%), as shown in Figure 2, indicating that the mechanism of the SiO_2_-film CMP could not be analyzed with only a chemical-dominant CMP. Thus, a mechanical-dominant CMP characteristic (i.e., the electrostatic force between ZrO_2_ abrasives and SiO_2_-film surface) in the CMP slurry was investigated as a function of the scavenger concentration, as shown in Figure 7b. Note that the zeta potential of the SiO_2_-film surface in the CMP slurry is represented by the zeta potential of the SiO_2_ particles in the CMP slurry. The attractive electrostatic force between ZrO_2_ abrasives and the SiO_2_-film surface in the CMP slurry decreased noticeably from 36.1 to 18.5 abs., when the scavenger concentration increased from 0 to 0.10 wt%. Thus, the SiO_2_-film surface polishing rate decreased with the scavenger concentration, since a higher attractive force between ZrO_2_ abrasives and the SiO_2_-film surface generally leads to a higher SiO_2_-film surface polishing rate [10], which was the opposite of the SiO_2_-film surface trend. This result indicates that up to a scavenger (i.e., EA) concentration of 0.10 wt%, the mechanism of the SiO_2_-film surface CMP follows a chemical-dominant CMP, since the dissolved O_2_ and radicals (i.e., OH^●^, HO_2_^●^, and O_2_^●−^) decreased with increasing scavenger concentration. However, the repulsive electrostatic force between the ZrO_2_ abrasives and the SiO_2_-film surface in the CMP slurry increased rapidly from 18.5 to 229.9 abs., when the scavenger concentration increased from 0.10 to 0.25 wt%. Thus, the SiO_2_-film surface polishing rate decreased with increasing scavenger concentration, since a higher repulsive force between the ZrO_2_ abrasives and the SiO_2_-film surface generally leads to a lower SiO_2_-film surface polishing rate [10]. This result means that, for a scavenger (i.e., EA) concentration above 0.10 wt%, the mechanism of the SiO_2_-film surface CMP is principally associated with a mechanical-dominant CMP rather than a chemical-dominant CMP, although the dissolved O_2_ and radicals (i.e., OH^●^, HO_2_^●^, and O_2_^●−^) decreased with increasing the scavenger concentration. Therefore, the mechanism of the SiO_2_-film surface polishing rate for a scavenger (i.e., EA) concentration ranging between 0 and 0.10 wt% was followed by a chemical-dominant CMP. On the other hand, the mechanism of the SiO_2_-film surface polishing rate for a scavenger (i.e., EA) concentration ranging between 0.10 and 0.25 wt% was conducted by a mechanical-dominant CMP. Thus, the SiO_2_-film surface polishing rate peaked at a specific scavenger concentration (i.e., 0.10 wt%).

For a practical application as a W-film surface CMP slurry, since the addition of a scavenger to a W-film surface CMP slurry could induce a corrosion of the W-film surface during CMP, although it significantly improves the abrasive dispersant stability in the slurry, a corrosion inhibitor with a protonated amine-functional group (i.e., C_4_H_8_N_2_O_3_: asparagine) of 0.1 wt% was additionally mixed with the W-film surface slurry including the scavenger (i.e., EA). The static etch rate was 0.75–0.85 nm/min, which was independent of the scavenger concentration, as shown in Figure S4. Thus, the addition of a corrosion inhibitor could prevent the generation of corrosion on the W-film surface. In addition, it was found that there was almost no difference in the W-film surface polishing rate depending on the scavenger concentration between the CMP slurry with and without a corrosion inhibitor, as shown in Figure S5. Therefore, the addition of a corrosion inhibitor to the W-film surface CMP slurry with a scavenger (i.e., EA) could achieve W-film surface CMP without the presence of corrosion on the W-film surface after CMP, as shown in Figure S6.

## 4. Conclusions

For advanced semiconductor devices such as AP, DRAM, and 3D NAND flash memory, the frequency of the W-film surface CMP has rapidly increased. In W-film surface CMP, the Fenton reaction should be utilized to enhance the W-film surface polishing rate by forming a nanoscale thick chemically oxidized WO_3_ layer on the W-film surface. However, the produced radicals (i.e., OH^●^, HO_2_^●^ and O_2_^●−^) via the Fenton reaction between the ferric–ionic catalyst and H_2_O_2_ directly degrades the magnitude of the abrasive dispersant stability in the W-film surface CMP slurry. As a solution, the addition of a scavenger to the W-film surface CMP slurry is essential, as it can significantly suppress the H_2_O_2_ decomposition rate and the amount of radicals can be noticeably reduced. Unlike a scavenger with a carboxyl functional group [10], a scavenger with phosphite (i.e., EA and PA) or phosphate (i.e., MPP and MAP) presented an incredible improvement in the abrasive dispersant stability in the W-film surface CMP. In particular, the improvement in the magnitude of the abrasive dispersant stability was remarkably enhanced with the scavenger concentration. Moreover, it was found that a higher improvement magnitude of the abrasive dispersant stability in the W-film surface CMP slurry was followed by EA, PA, MPP, and MAP, since a lower sequence of a H_2_O_2_ decomposition rate was performed by EA, PA, MPP, and MAP. However, the addition of a scavenger with double-negatively charged phosphite ions could induce corrosion on the W-film surface via a chemical reaction between WO_3_ and the ionized EA (i.e., (C_2_H_6_O_7_P_2_)^2−^) so that the W-film surface polishing rate decreased linearly and significantly with increasing scavenger concentration. In addition, the amount of dissolved O_2_ to form WO_3_ on the W-film surface via the Fenton reaction to decompose H_2_O_2_ significantly decreased with increasing scavenger (i.e., EA) concentration. Thus, due to the presence of corrosion and the reduction of the dissolved O_2_ amount by adding a scavenger to the W-film surface CMP slurry, the W-film surface polishing rate decreased notably with increasing scavenger concentration. These results indicate that the mechanism of the W-film surface CMP depending on the scavenger concentration in the W-film surface CMP slurry was principally determined by chemical-dominant CMP.

The addition of a scavenger to the W-film surface CMP slurry could influence the SiO_2_-film surface polishing rate depending on the scavenger concentration as well as the mechanism of the SiO_2_-film surface CMP. Since the amount of dissolved O_2_ and radicals (i.e., OH^●^, HO_2_^●^ and O_2_^●−^) in the W-film surface CMP slurry via the Fenton reaction decreased significantly with increasing scavenger concentration, chemical oxidation via chemical reaction between the hydrolyzed Si(OH)_4_ surface, dissolved O_2,_ and radicals was notably enhanced with increasing scavenger concentration. As a result, at the scavenger concentration between 0 and 0.10 wt%, the SiO_2_-film surface polishing rate increased noticeably with the scavenger concentration, which was mainly determined by chemical-dominant CMP. Otherwise, above the scavenger concentration of 0.10 wt%, since the repulsive electrostatic force between the negatively charged ZrO_2_ abrasive and SiO_2_-film surface increased rapidly with the scavenger concentration, the SiO_2_-film surface decreased significantly with increasing scavenger concentration. As a result, the SiO_2_-film surface polishing rate peaked at a specific scavenger concentration, indicating that the mechanism of the SiO_2_-film surface CMP was chemical dominant as well as mechanical-dominant CMP. The negative effect of the presence of corrosion on the W-film surface could be eliminated by adding a corrosion inhibitor with a carboxyl functional group without an additional decrease in the W- and SiO_2_-film surface polishing rate. Therefore, the addition of a scavenger with phosphite (i.e., EA and PA) or phosphate (i.e., MPP and MAP) to the W-film surface CMP slurry could remarkably improve the abrasive dispersant stability in the W-film surface CMP slurry for practical CMP applications in semiconductor devices. Further studies would be necessary for another ferric–ionic catalyst for the Fenton reaction such as ammonium iron(III) citrate, ammonium iron(III) oxalate trihydrate, iron(III) chloride hexahydrate, iron(III) sulfate hydrate, and Potassium ferrocyanide. In addition, the improvement effect of the abrasive dispersant stability using these catalysts, including a scavenger with phosphite or phosphate ions, is necessary for further research. Therefore, the chemical design of a scavenger in the slurry for the W-film surface CMP via the Fenton reaction would be key to achieving a higher W- and a proper SiO_2_-film polishing rate as well as a longer abrasive dispersant stability.

## Data Availability

Data can be available upon request from the authors.

## Supplementary Materials

The following are available online at https://www.mdpi.com/article/10.3390/nano11123296/s1: Figure S1: Dependencies of the ZrO_2_ abrasive stability in the W-film-surface slurry mixed with the oxidant (i.e., H_2_O_2_) as a function of the scavenger type and concentration. The H_2_O_2_ decomposition-rate as a function of the progress time after mixing H_2_O_2_ into the W-film-surface slurries with (a) EA, (b) PA, (c) MPP and (d) MAP.; Figure S2: H_2_O_2_ decomposition rate depending on scavenger type and mole concentration.; Figure S3: Hardness of W-, TiN-, SiO_2_-, and poly-Si-film.; Figure S4: Effect of corrosion inhibitor on suppressing the degree of corrosion (i.e., static etch rate) and surface morphology. Background SEM images show the presence and degree of corrosion on the W-film-surface after dipping the W-film into the CMP slurry including a scavenger (i.e., EA). The lateral profile of image contrast on the SEM images corresponded to the degree of corrosion. The addition of the corrosion inhibitor (i.e., asparagine) in the W-film-surface slurry with a scavenger (i.e., EA) suppressed remarkably the corrosion degree (i.e., static etch rate); Figure S5: Effect of a corrosion inhibitor (i.e., asparagine) on CMP performance of W-film-surface slurries, including a scavenger (i.e., EA). (a) Dependencies of W-and SiO_2_-film-surface polishing rate on the scavenger concentration for the W-film-surface slurry including both scavenger (i.e., EA) and corrosion inhibitor (i.e., asparagine) and (b) magnified W- and SiO_2_-film-surface polishing rates depending on the scavenger concentration from (a). The addition of the corrosion inhibitor (i.e., asparagine) in the W-film-surface slurry with a scavenger (i.e., EA) showed no change of W- and SiO_2_-film-surface polishing rates; Figure S6: Potentiodynamic polarization curves of the W-film surface depending on the scavenger (i.e., EA) concentration and corrosion inhibitor (i.e., asparagine). The upper inset showed none of the surface chemical oxidation. The lower inset represented both corrosion potential (i.e., E_corr_) and corrosion current (i.e., I_corr_), which was independent of the scavenger concentration.
