# Supplementary material for: Scavenger with Protonated Phosphite Ions for Incredible Nanoscale ZrO2-Abrasive Dispersant Stability Enhancement and Related Tungsten-Film Surface Chemical–Mechanical Planarization
Source: Nanomaterials (Basel). 2021 Dec 4;11(12):3296. doi: 10.3390/nano11123296 (PMC8706925; doi:10.3390/nano11123296)
Supplement: Supplementary file 1 [file nanomaterials-11-03296-s001.zip › nanomaterials-1467815-supplementary.pdf]

Supplementary Materials

Scavenger with Protonated Phosphite Ions for Incredible Nanoscale ZrO_2-Abrasive Dispersant stability Enhancement and Related Tungsten-Film Surface Chemical–Mechanical Planarization

Seong-In Kim [1], Gi-Ppeum Jeong [2], Seung-Jae Lee [2], Jong-Chan Lee [1], Jun-Myeong Lee [1], Jin-Hyung Park [3], Jae-Young Bae [4] and Jea-Gun Park [1,2,*]

[1] Department of Nanoscale Semiconductor Engineering, Hanyang University, Seoul 04763, Korea; rlatjddls25805@naver.com (S.-I.K.); student.jongchan@gmail.com (J.-C.L.); kjunom200@naver.com (J.-M.L.)
[2] Department of Electronic Engineering, Hanyang University, Seoul 04763, Korea; bono23231@naver.com (G.-P.J.); ltwobooml@naver.com (S.-J.L.)
[3] UB Materials Inc., Gyeonggi-do 17162, Korea; parkjinhyung@gmail.com
[4] Department of Energy Engineering, Hanyang University, Seoul 04763, Korea; jaeyoungb@daum.net
* Correspondence: parkjgl@hanyang.ac.kr

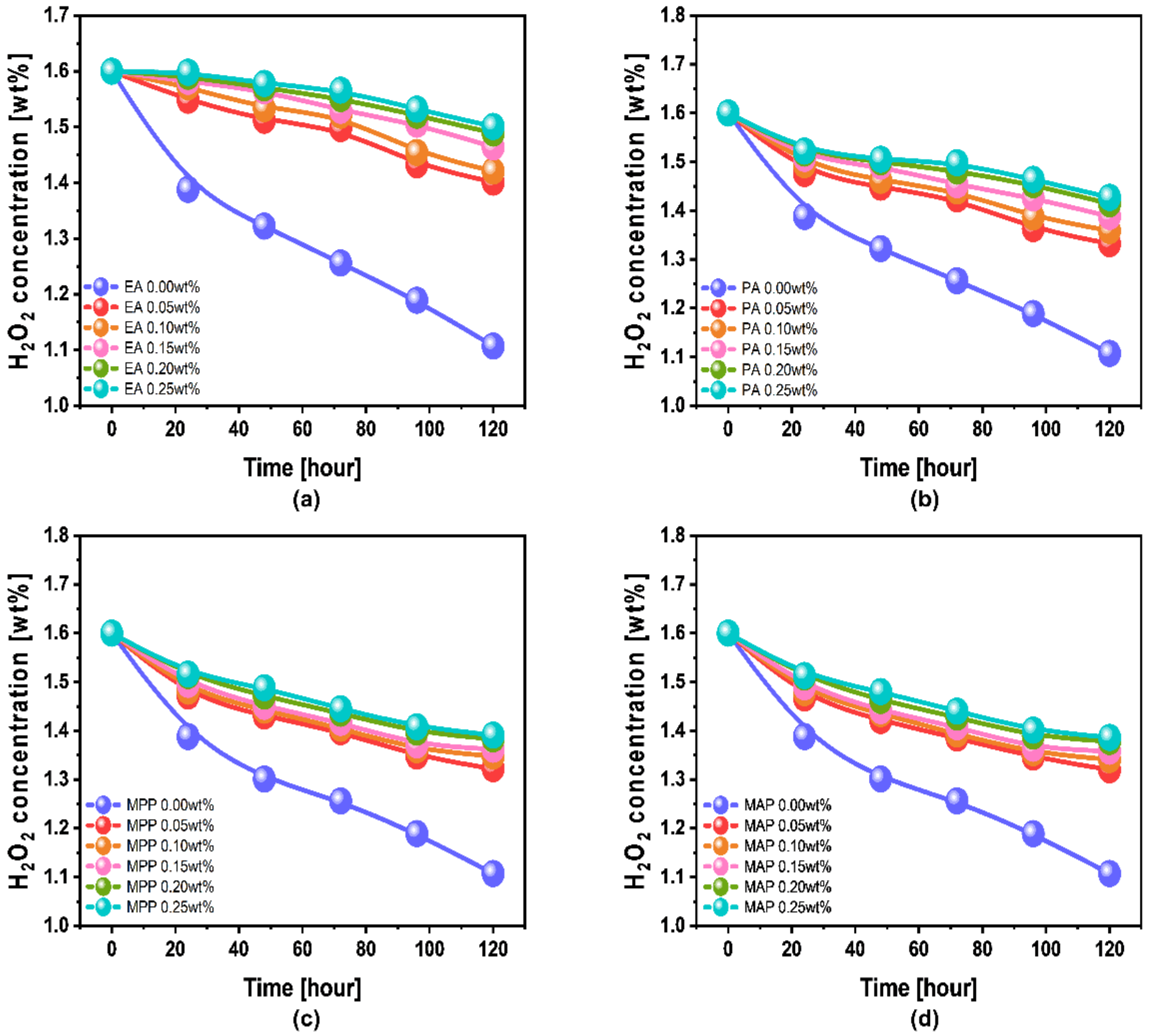

Figure S1. Dependencies of the ZrO_2 abrasive stability in the W-film-surface slurry mixed with the oxidant (i.e., H_2O_2) as a function of the scavenger type and concentration. The H_2O_2 decomposition-rate as a function of the progress time after mixing H_2O_2 into the W-film-surface slurries with (a) EA, (b) PA, (c) MPP and (d) MAP.

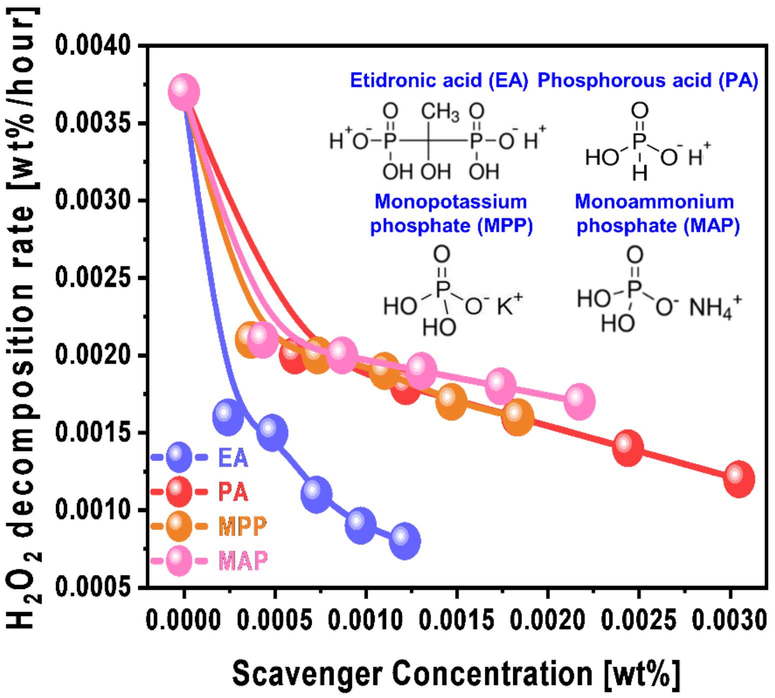

Figure S2. H_2O_2 decomposition rate depending on scavenger type and mole concentration.

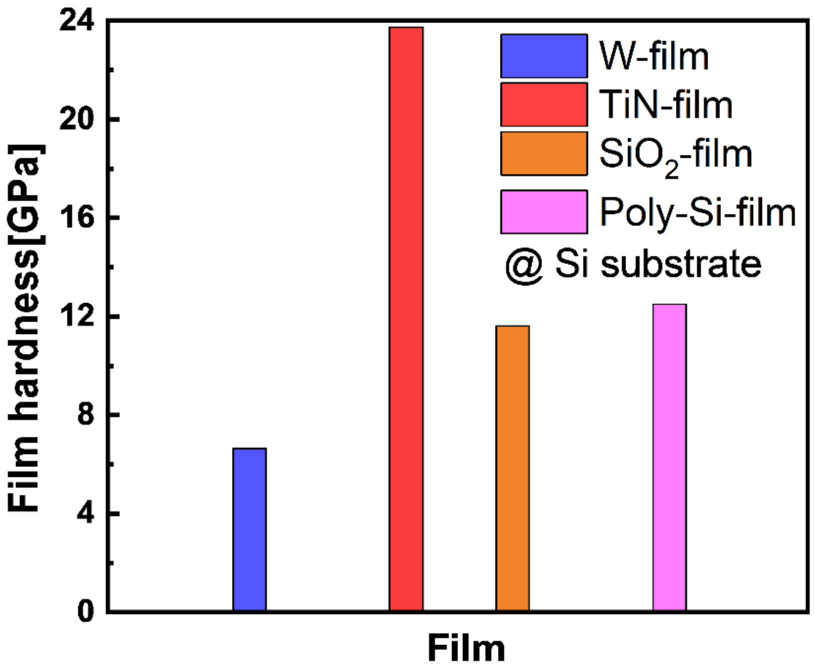

Figure S3. Hardness of W-, TiN-, SiO_2-, and poly-Si-film.

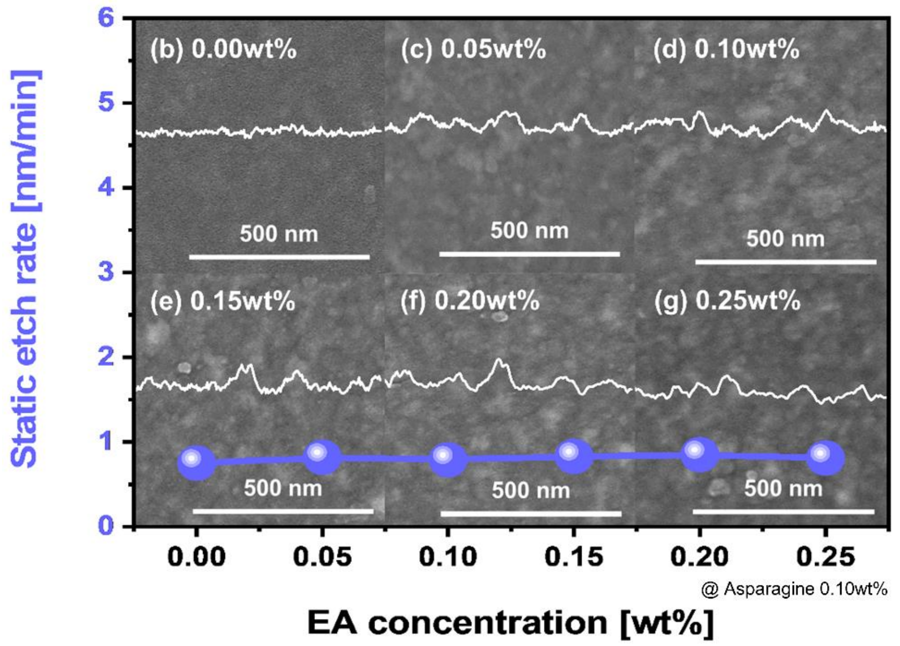

Figure S4. Effect of corrosion inhibitor on suppressing the degree of corrosion (i.e., static etch rate) and surface morphology. Background SEM images show the presence and degree of corrosion on the W-film-surface after dipping the W-film into the CMP slurry including a scavenger (i.e., EA). The lateral profile of image contrast on the SEM images corresponded to the degree of corrosion. The addition of the corrosion inhibitor (i.e., asparagine) in the W-film-surface slurry with a scavenger (i.e., EA) suppressed remarkably the corrosion degree (i.e., static etch rate).

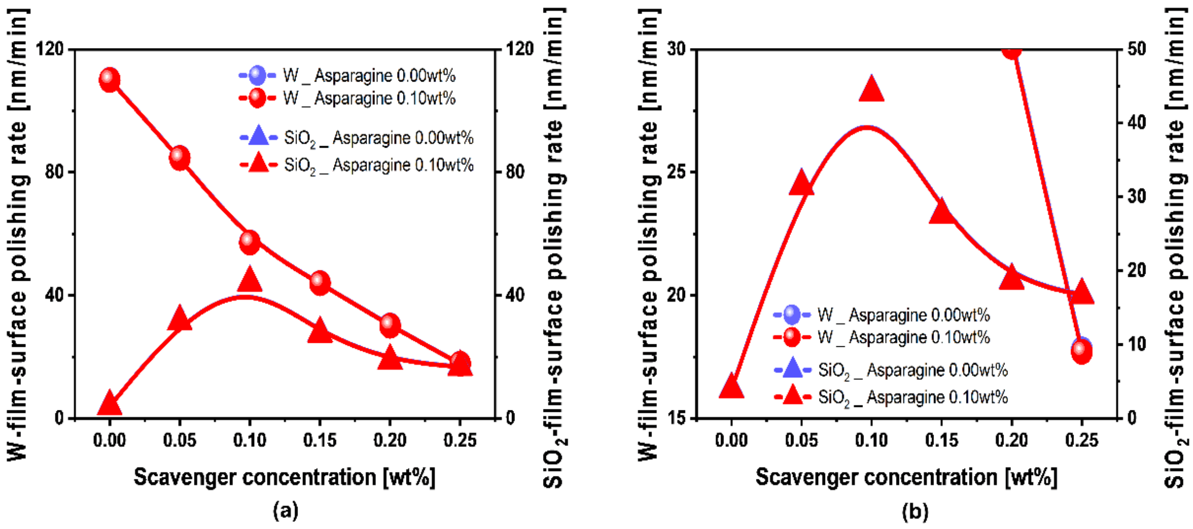

Figure S5. Effect of a corrosion inhibitor (i.e., asparagine) on CMP performance of W-film-surface slurries, including a scavenger (i.e., EA). (a) Dependencies of W-and SiO_2-film-surface polishing rate on the scavenger concentration for the W-film-surface slurry including both scavenger (i.e., EA) and corrosion inhibitor (i.e., asparagine) and (b) magnified W- and SiO_2-film-surface polishing rates depending on the scavenger concentration from (a). The addition of the corrosion inhibitor (i.e., asparagine) in the W-film-surface slurry with a scavenger (i.e., EA) showed no change of W- and SiO_2-film-surface polishing rates.

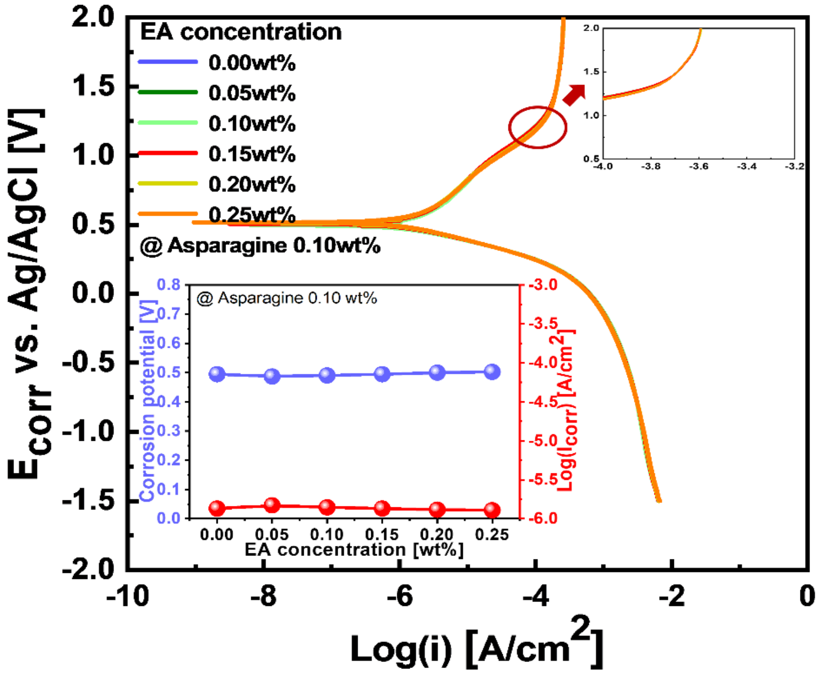

Figure S6. Potentiodynamic polarization curves of the W-film surface depending on the scavenger (i.e., EA) concentration and corrosion inhibitor (i.e., asparagine). The upper inset showed none of the surface chemical oxidation. The lower inset represented both corrosion potential (i.e., E_{corr}) and corrosion current (i.e., I_{corr}), which was independent of the scavenger concentration.
